# Supplementary material for: A neotropical perspective on the uniqueness of the Holocene among interglacials
Source: Nat Commun. 2023 Nov 16;14:7404. doi: 10.1038/s41467-023-43231-0 (PMC10654573; doi:10.1038/s41467-023-43231-0)
Supplement: Supplementary file 1 — Supplementary Information [file 41467_2023_43231_MOESM1_ESM.pdf]

Supplementary Material for

A Neotropical Perspective on the Uniqueness of the Holocene among Interglacials

Schiferl, J., Kingston, M. , Åkesson, C.M., Valencia B.G., Rozas-Davila A., McGee, D., Woods, A., Chen, C.Y. , Hatfield R.G., Rodbell, D.T., Abbott, M.B., Bush, M.B.

Glaciation around Lake Junín.

Wright et al. [1] mapped the moraines surrounding Lake Junín. In general, the glaciated portion of the landscape lies above 4200 m, with outwash features forming fans down to c. 4100 m. Thus, the lake, which lies at 4085 m, was surrounded by an unglaciated margin that was commonly 4 - 10 km wide (Fig. S1).

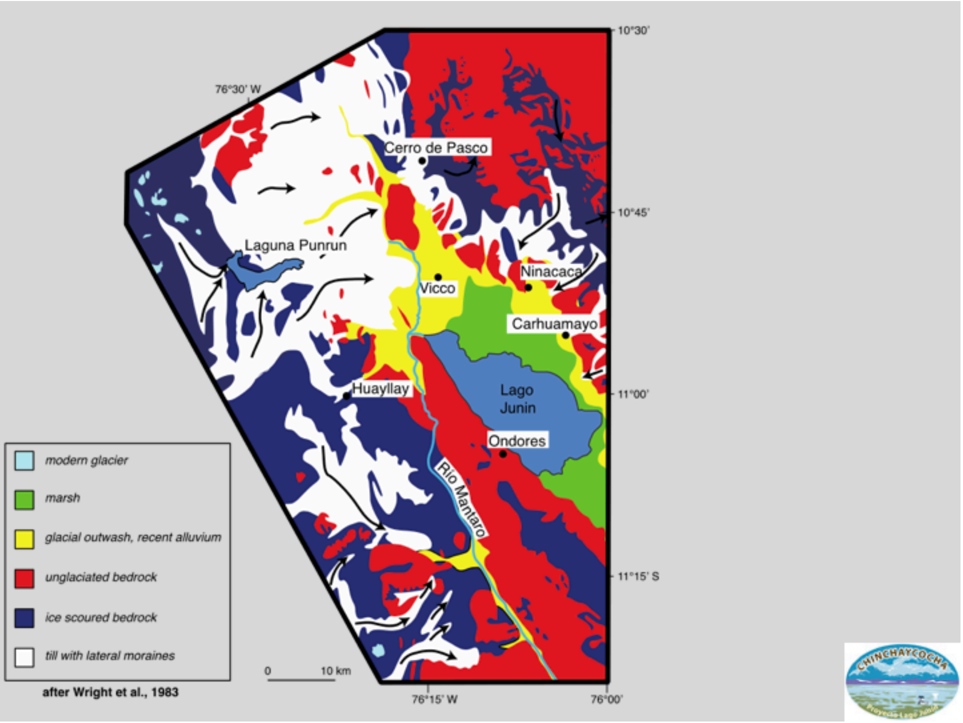

Fig. S1: Sketchmap showing the extent of glaciation surrounding Lake Junín. Modified from [1].

RESULTS

The chronology developed for core JU C15-1 yielded an age model with a fairly constant rate of sedimentation (Fig. S2).

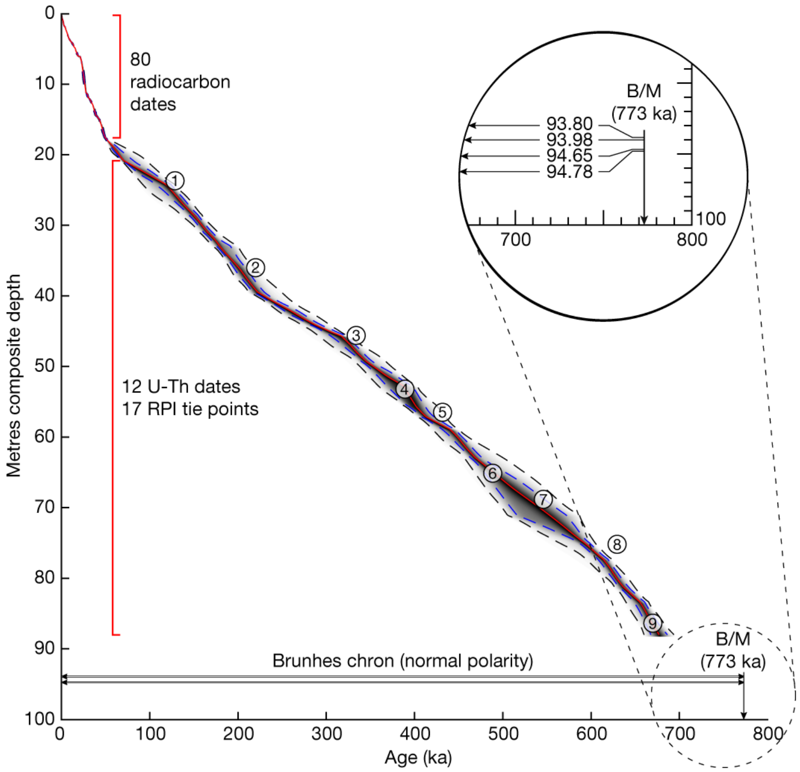

Fig S2: The chronology for core JU C15-1 from Lake Junín, Peru. The age–depth relationship is based on 80 radiocarbon dates [2] (<17 meters composite depth (mcd)), 12 U-Th-dated intervals of authigenic calcite from five carbonate intervals between about 21 and 71 mcd [3] and 17 palaeomagnetic tie points [4]. The red line is the mean age model; purple and black dashes represent the 1 sigma and 2 sigma uncertainties around the mean, respectively. Four arrows mark the depth of four samples that yielded normal polarity (depths shown in the inset along the age of the Brunhes–Matuyama (B/M) reversal boundary) and are younger than 773 ka [5]

(see Methods). Numbers 1–9 are tie points used as the age model; tie points are for illustration only and were not used in the generation of this radiometric and palaeomagnetic age model [4]. Figure from Rodbell et al.[6]).

The Lake Junín fossil pollen analysis consisted of 508 samples with well-preserved pollen, in which 445 of 508 (88%) samples reached either 300 terrestrial pollen grains or 2000 microspheres (samples). Pollen diversity was high for this elevation with 213 morphotypes recognized, of which 76% (161 morphotypes) were identified to family or genus. Unidentified grains averaged ~1 (maximum 10) % and broken grains averaged ~5% (maximum 28%) of the pollen sum.

Pollen

Most glacials were characterized by very low pollen influx (i.e. 10-80 grains per cm^2 per yr), whereas in interglacials values ranged as high as 8000 grains per cm^2 per yr (Fig. 2). The log pollen influx and the amount of puna grassland closely approximated the LR04 curve for marine isotopic change through the last 650,000 years (Fig. 5). The earlier interglacials (MIS 15, 13, and 11) had markedly higher pollen influxes than the later ones (Fig. 2).

During glacials, the most important pollen taxa were *Alnus* and members of the Puna shrubland (Table S1), primarily comprised of Asteraceae and *Polylepis* (Fig. S3). Although most abundant during glacials, puna shrubland taxa also occurred during cool or moist periods within interglacials. Contrastingly, *Alnus* was almost always linked to very low local productivity, indicated by low pollen influx. Another group that had most of its peaks during times of low

pollen influx was lower montane forest (Fig. 1). This group was diverse, but included taxa that today would generally be found below 2800 m elevation, e.g. Moraceae-Urticaceae, *Celtis,* *Trema*, and *Cecropia* (Fig. S3).

Table S1: Pollen taxa assigned to main vegetation groups.

**Puna grassland**	**Puna shrubland**	**Upper montane forest**	**Lower montane forest**
Poaceae	cf. *Artemisia* (Asteraceae)	*Alnus* (Betulaceae)	Iresine (Amaranthaceae)
Zea mays (Poaceae)	Asteraceae	*Podocarpus* (Podocarpaceae)	Amaranthus (Amaranthaceae)
Plantago (Plantaginaceae)	Asteraceae Morphotypes 1-18	*Hedyosmum* (Chloranthaceae)	*Juglans* (Juglandaceae)
Brassicaceae	cf. *Cichorium/Prenanthes* (Asteraceae)	*Weinmannia* (Cunoniaceae)	*Acalypha* (Euphorbiaceae)
cf. *Lepidium* (Brassicaceae)	cf. *Jungia/Perezia* (Asteraceae)	Moraceae/Urticaceae	*Alchornea* (Euphorbiaceae)
Apiaceae (others)	*Polylepis* (Rosaceae)	*Miconia* (Melastomataceae)	Malvaceae
cf. *Apium* (Apiaceae)	*Ambrosia* (Asteraceae)	Melastomatac./.Combretac.	*cf. Herissantia* (Malvaceae)
Caryophyllaceae	Amaranthaceae	*Morella* (Myricaceae)	*Cecropia* (Urticaceae)
Valeriana (Caprifoliaceae)	*Hypericum* (Hypericaceae)	*Myrsine* (Primulaceae)	*Celtis* (Cannabaceae)
Ephedra (Ephedraceae)	Ericaceae	*Rapanea* (Primulaceae)	*Trema* (Cannabaceae)
Descurainia (Brassicaceae)	*Desmoscelis* (Melastomataceae)	*Vallea* (Elaeocarpaceae)	*Croton* (Euphorbiaceae)
Gentianella (Gentianaceae)	Solanaceae	Myrtaceae	*Sebastiania* (Euphorbiaceae) sum
Stangea (Caprifoliaceae)	*Hydrocotyle* (Araliaceae)	*Bocconia* (Papaveraceae)	*Sebastiania* cf. *Schottiana* (Euphorbiaceae)
Ranunculus (Ranunculaceae)	*Cleome* (Cleomaceae)	Rubiaceae	*Sebastiania* cf. *Pachystachys*
cf. *Anemone* (Rununculaceae)	Lamiaceae	*Galium (*Rubiaceae)	Fabaceae cf. *Senna*
Rununculaceae spp.	cf. *Puya* (Bromeliaceae)	*Dodonaea* (Sapindaceae)	Fabaceae (C) *Dialium*
Castilleja (Orobanchaceae)	*Thalictrum* (Ranunculaceae)	*Sambucus (Adoxaceae)*	Fabaceae (Caesalpinioideae)
*Gentiana (*Gentianaceae)	*Alternanthera* (Amaranthaceae)	*Salix* (Salicaceae)	Fabaceae (Mimosoideae)
Gunnera (Gunneraceae)	Euphorbiaceae (others)	*Ilex* (Aquifoliaceae)	Fabaceae (Papilionoideae)
Rumex (Polygonaceae)	*Euphorbia* (Euphorbaceae)	*Styloceras* (Buxaceae)	*Euplassa* (Proteaceae)
Cactaceae	*Symplocos* (Symplocaceae)	*Buxus* (Buxaceae)	*Roupala* (Proteaceae)
	cf. *Pediomelum* Fabaceae (P)		*Begonia* (Begoniaceae)
	*Anthurium (*Araceae)		*Ficus* (Moraceae)
	Coriaria (Coriariaceae)		Loranthaceae

	Campanulaceae		*Dendropanax* (Araliaceae)
	Anacardiaceae		*Schefflera* (Araliaceae)
	*Balbisia (*Vivianiaceae)		*Sloanea* (Elaeocarpaceae)
			Protium (Burseraceae)
			*Pinzona (*Dilleniaceae)
			Aphelandra (Acanthaceae)
			cf. *Turnera* (Passifloraceae)
			Spermacoce (Rubiaceae)
			Psyllocarpus (Rubiaceae)
			Trichilia (Meliaceae)
			Tabebuia (Bignoniaceae)
			Hamelia (Rubiaceae)
			Isertia (Rubiaceae)
			Callicarpa (Lamiaceae)
			Mauritiella (Arecaceae)
			*Phragmotheca (*Malvaceae)
			Eschweilera (Lecythidaceae)

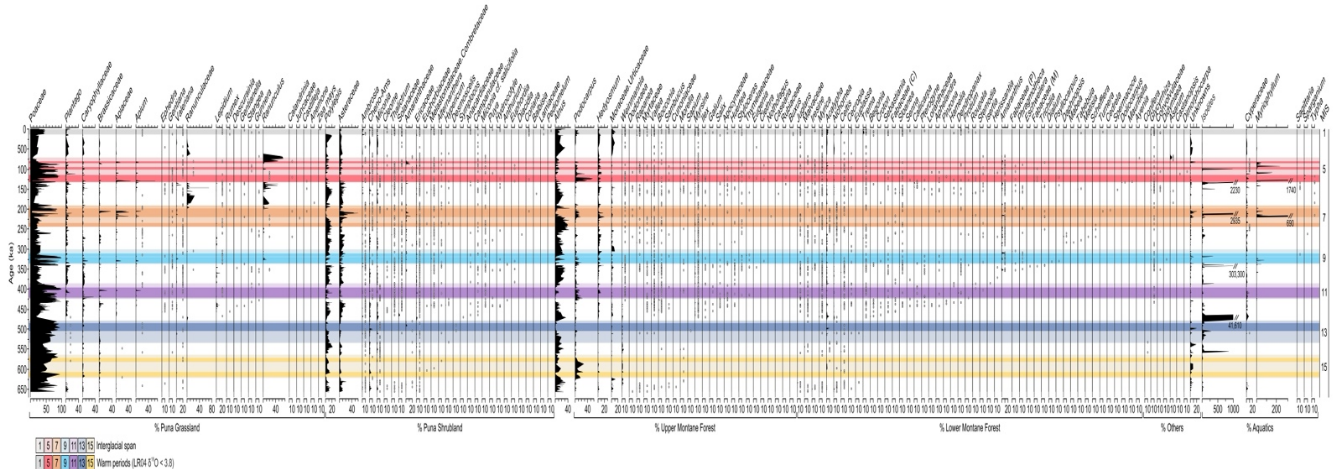

Fig. S3: Percentile diagram of all morphotypes encountered in the fossil pollen reconstruction of Lake Junin, Peru. Color coding corresponds to interglacial marine isotope stages of 1, 5, 7, 9, 11, 13 and 15.

The spores of *Isoëtes*, an aquatic quillwort, exhibited isolated, massive, peaks of abundance that reached 303,300% of the terrestrial pollen sum at 327 ka and 41,610% at 464 ka (Fig. S3). *Isoëtes* would have grown in the shallows of the lake margin or in very marshy ground adjacent to the lake and would have been susceptible to prolonged ice cover [7].

Interglacials were marked by increases in the pollen of upper Andean forest taxa, especially *Podocarpus* and *Weinmannia*. Although Poaceae was very abundant throughout the record, the highest peaks of Poaceae occurred during interglacials intercalated with peaks of *Podocarpus*. Large peaks of the aquatic taxon *Myriophyllum* occurred during MIS 5 at 111.7 and within MIS 7 at 201.5 ka with ~17x and 7x the terrestrial pollen sum, respectively. Maize pollen was recovered from two consecutive Holocene samples at 1.8 and 1.2 ka (Fig. S2).

The sample scores on Axis 1 of the DCA, did not result in a clean separation of any glacial from interglacials (Fig. S3). In general, interglacials had neutral or negative scores, as did the early glacials (MIS 16, 14, 12, and 10), while the later glacials (MIS 2, 6, and 8), had predominantly positive scores (Fig. S4). Species scores at the negative extreme of DCA Axis 1 were characterized by Poaceae, *Podocarpus*, and arboreal elements of the upper Andean forest (Fig. S3). At the positive extreme of Axis 1 were *Ranunculus*, other Ranunculaceae, and *Gunnera.* The negative scores of MIS 1 samples on Axis 2 largely segregated it from other interglacials, although some overlap was evident with two samples from MIS 13.

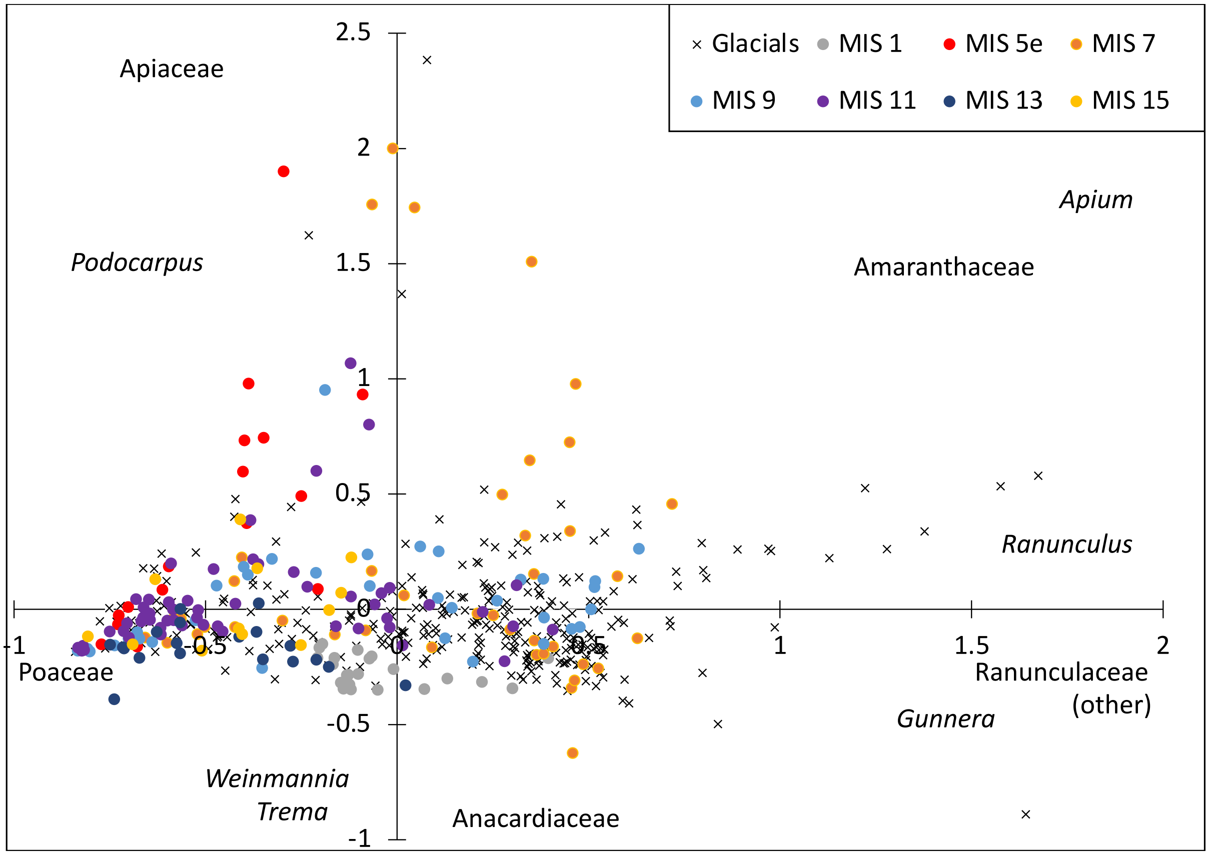

Fig S4: Results of the Detrended Correspondence Analysis of fossil pollen samples from Lake Junín, Peru. Also shown are the plants characterizing the extremes of each axis.

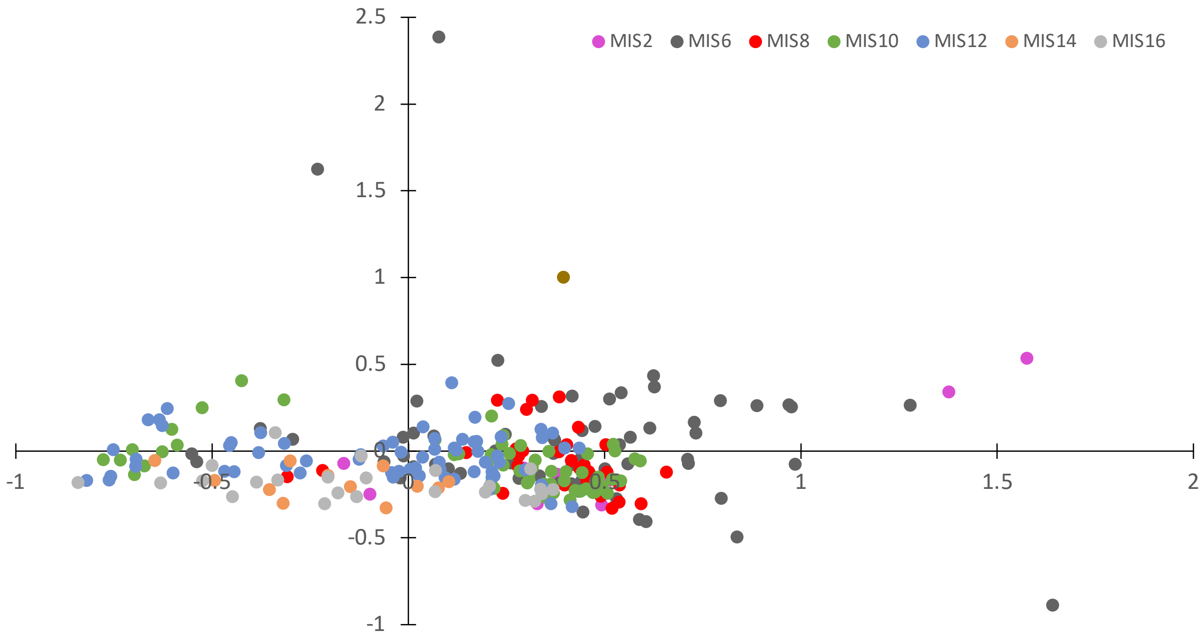

Fig. S5: Results of the Detrended Correspondence Analysis of fossil pollen samples from Lake Junín, Peru, showing just glacial aged samples. Species scores are the same as in Fig. S4. Axis 1 versus Axis 2 DCA scores for fossil pollen data, run with all samples, but only plotting samples attributed to glacial events.

Charcoal

Charcoal was found in a small minority of samples, with MIS 9, MIS 5 and MIS 1 being the only periods where it was documented in more than one sample (Table S2, Fig. 6). The most consistent occurrence of charcoal and the highest values of charcoal abundance occurred in MIS 1 (Table S2). Of the 17 samples that contained charcoal but fell outside the MIS zones listed in Table S2, six occurred between 16.6 and 11.7 ka and seven occurred within MIS 5 a-c, leaving only 3 samples with charcoal in all other periods.

Table S2: The number and proportion of samples within each interglacial containing charcoal.

	MIS 1	MIS 5	MIS 7	MIS 9	MIS 11	MIS 13	MIS 15	Others
n (%) with charcoal	21 (95)	9 (24)	1 (2)	5 (15)	1 (2)	0	1 (5)	17 (2)
Samples in interglacial	22	37	40	28	53	18	22	520

The pollen signatures of glacials and interglacials led to overlapping scores on the DCA ordination (Fig. S4) reflects a combination of factors: 1) that this area was always some version of a grassland, and we are unable to differentiate Poaceae pollen below the family level, 2) long-distance transport of pollen, especially during times of very low local pollen productivity, and 3) the climatic complexity of both glacials and interglacials in which warm or cold, respectively, times could blur the broader climatic patterns. Nevertheless, warmer, more productive assemblages had negative scores on Axis 1, while the glacials generally had neutral or positive scores. The taxa characterizing the negative extreme of Axis 1 included Poaceae and Podocarpus. The high relative abundance of Poaceae during interglacials primarily indicates its local dominance and that when so abundant its percentage occurrence is no longer diluted by long-distance pollen transportation. In this vegetation record, the samples that were statistically most distinct from the interglacials belonged to MIS 2, 6, and 8. The DCA scores for these events had the most positive scores on Axis 1 (Fig. S5). Ranunculaceae pollen characterized the positive extreme of Axis 1. These wetland herbs probably grew in marshes flanking the lake. The proximity of ice to the lake during glacial stages is attested by complex moraine fields representing multiple advances terminating close to the modern shoreline [8]. Thus, upslope of the lake would have been largely ice-covered and unproductive in terms of pollen, while the lake shoreline would have been marshes giving way to a glacial foreland.

1 Wright, H. E., Jr. Late-Pleistocene glaciation and climate around the Junin Plain, central Peruvian Andes. *Geografiska Annaler* **65A**, 35-43 (1983).
2 Woods, A. *et al.* Andean drought and glacial retreat tied to Greenland warming during the last glacial period. *Nature Communications* **11**, 1-7 (2020).
3 Chen, C. Y. *et al.* U-Th dating of lake sediments: Lessons from the 700 ka sediment record of Lake Junín, Peru. *Quaternary Science Reviews* **244**, 106422 (2020).
4 Hatfield, R. G. *et al.* Paleomagnetic constraint of the Brunhes age sedimentary record from Lake Junín, Peru. *Frontiers in Earth Science* **8**, 147 (2020).
5 Channell, J. E., Hodell, D. A., Singer, B. S. & Xuan, C. Reconciling astrochronological and 40Ar/39Ar ages for the Matuyama-Brunhes boundary and late Matuyama Chron. *Geochemistry, Geophysics, Geosystems* **11** (2010).
6 Rodbell, D. T. *et al.* 700,000 years of tropical Andean glaciation. *Nature* **607**, 301-306 (2022).
7 Cleef, A. M. *The vegetation of the paramos of the Colombian Cordillera Oriental.*, (University of Amsterdam, Dissertationes Botanicae 61, 1-320 (1981).
8 Smith, J. A., Seltzer, G. O., Farber, D. L., Rodbell, D. T. & Finkel, R. C. Early local last glacial maximum in the tropical Andes. *Science* **308**, 678-681 (2005).
